# Health professions education and unprofessional behaviour in the global south: a scoping review of conceptions, theoretical frameworks, and prevalence

**DOI:** 10.1186/s12909-025-08318-w

**Published:** 2025-12-29

**Authors:** Lunelle Pienaar, Desmond Kuupiel, Jacqueline van Wyk

**Affiliations:** 1 Department of Health Sciences Education, Faculty of Health Sciences, University of Cape Town, Observatory, Cape Town, 7935 South Africa; 2 Discipline of Public Health Medicine, School of Nursing and Public Health, University of KwaZulu-Natal, Durban, 4001 South Africa

**Keywords:** Professionalism, Global south, Low- and Middle-Income countries (LMICs), Academic dishonesty, Bullying and harassment, Clinical and ethical misconduct, Disrespect and power abuse, Neglect of professional responsibilities

## Abstract

**Background:**

Understanding how unprofessionalism is interpreted and enacted in low- and middle-income countries is essential for developing health professionals that are contextually and socially grounded. The scoping review explores and maps existing scientific evidence on unprofessional behaviour in health professions education from a Global South perspective. The review was informed by the research question: How is unprofessional behaviour defined, conceptualised, and framed theoretically in health professions education within the Global South?

**Methods:**

Using a scoping review, we retrieved 382 articles, of which 14 articles were published between 2004 and 2024. The articles were retrieved across PubMed/MEDLINE, Scopus, Web of Science, EBSCOhost (Academic Search Complete, Health Source, and PsycINFO) databases, and supplemented by Google Scholar.

**Results:**

The studies emerged from 10 countries, with the majority conducted in the United Arab Emirates (21.4%), followed by Saudi Arabia and Thailand with 14.3% each, and other countries each contributing 7.1% of the total studies. The highest number of studies was published in 2017, 2020, and 2023 (14.3% each). Most study designs were cross-sectional (71.5%), while qualitative studies accounted for 21.4%, and mixed methods were 7.1%. The study populations predominantly consisted of medical students (64.4%), followed by residents (14.3%), and smaller groups including multi-disciplinary students (Medicine, Pharmacy, Nursing), clinicians and medical students, and clinical faculty members and medical students with 7.1% each. The studies were conducted across academic and clinical settings (50.0%), with others focusing solely on clinical environments (28.6%), preclinical settings (14.3%), and a clinical and surgical training environment (7.1%). Five key themes emerged: Academic Dishonesty and Integrity Violations, Bullying and Harassment, Clinical and Ethical Misconduct, Disrespect and Power Abuse, and Neglect of Professional Responsibilities.

**Conclusions:**

The study findings draw attention to the need for theoretical engagement and institutional reforms that reflect the realities of educational and clinical training environments in low- and middle-income countries.

**Supplementary Information:**

The online version contains supplementary material available at 10.1186/s12909-025-08318-w.

## Background

Professionalism is a fundamental pillar of health professions education and practice. It encompasses a set of values, behaviours, and relationships that underpin the trust the public places in healthcare professionals [1–3]. However, despite efforts to integrate professionalism into curricula, unprofessional behaviour among students and practitioners continues to be a global concern with potential implications for patient safety, institutional integrity, and the development of professional identity [3–5].

In many health professions education contexts, unprofessional behaviour has been linked to future disciplinary action, suggesting a need for early identification and intervention. For instance, Teherani et al. found that medical students who demonstrated issues such as poor reliability, resistance to feedback, and low motivation were more likely to face licensing board actions in later professional life [6]. Despite such findings, there is no consensus on what constitutes unprofessional behaviour, with interpretations often shaped by institutional norms, cultural values, and contextual factors [7, 8].

Most of the existing literature and conceptual frameworks related to unprofessional behaviour originate from high-income countries in North America, Europe, and Australia. These studies often frame unprofessionalism using Western sociocultural and institutional lenses [9, 10]. However, the understanding and demonstration of professionalism depend on the context, shaped by history, politics, and culture, and thus cannot be universally transferred without alteration. Evidence from the Global South remains sparse, fragmented, and underrepresented, despite differing contextual realities, hierarchies, and cultural expectations in educational and clinical settings [11, 12].

In regions such as Africa, Asia, Latin America, and the Middle East, the occurrence of unprofessional behaviour is often shaped by resource constraints, hierarchical clinical environments, and limited reporting mechanisms [8, 13]. For example, students in South Africa and Iran have reported witnessing frequent patient mistreatment and ethical lapses, often without institutional response or support [8, 11]. Furthermore, there is a lack of clarity regarding how professionalism is taught, understood, and operationalised in these settings, making it difficult to compare findings or develop regionally relevant interventions [14].

Despite the clear importance of addressing unprofessional behaviour in health professions education, no comprehensive review has yet synthesised how unprofessionalism is defined, conceptualised, theorised, or measured across the Global South. Without such a synthesis, educators and policy-makers risk importing interventions or frameworks that may not align with local realities or educational structures. There is also a need to map the prevalence and types of unprofessional behaviours reported within different disciplines such as medicine, nursing, and allied health, particularly in clinical and academic environments across low- and middle-income countries.

Therefore, this scoping review set out to explore and map the existing scientific evidence on unprofessional behaviour in health professions education in the Global South. Specifically, the review seeks to: identify how unprofessional behaviour is defined and conceptualized in health professions education; explore the theoretical frameworks used to examine unprofessionalism in these contexts; examine the prevalence, patterns, and settings in which unprofessional behaviour occurs; and understand how instances of unprofessional behaviour are reported, managed, or addressed hoping to highlight knowledge gaps, and offer direction for future research and educational reform tailored to the needs of health professions education in the Global South.

## Methods

This scoping review followed the methodological framework proposed by Arksey and O’Malley and further refined by Levac et al. [15, 16], and reported in keeping with the Preferred Reporting Items for Systematic Reviews and Meta-analyses modified for Scoping Reviews checklist [17]. The framework includes five key mandatory stages: (i) identifying the research question; (ii) identifying relevant studies; (iii) selecting studies; (iv) charting the data; (v) collating, summarising, and reporting the results [15, 16]. Clinical trial number: not applicable.

### Identifying the research question

This scoping review’s main question was: How is unprofessional behaviour conceptualised, and framed theoretically in health professions education within the Global South? This question is structured according to the Population, Concept, and Context (PCC) framework recommended for scoping reviews, as summarised in Table 1 below.

**Table 1:** Population, Concept, and context framework for this scoping review’s question

Element	Description
Population	This included: students enrolled in health professions education programs (e.g., Medicine, Nursing, Occupational Therapy, Physiotherapy, Pharmacy, etc.) as well as educators, faculty members, and clinical preceptors involved in training these students
Concept	This included the following: • Descriptions and conceptions of unprofessional behaviour • Theoretical and conceptual frameworks explaining unprofessionalism • Prevalence and patterns of unprofessional behaviour • Reporting, management, and response mechanisms
Context	This referred to the following: • Health professions education in the Global South (including countries in Africa, Asia, Latin America, and the Middle East) • Clinical, academic, and community-based training settings

To explore this comprehensively, we formulated the following sub-questions:

1. What conceptions of unprofessional behaviour have been documented in the context of health professions education in the Global South?
2. What theoretical frameworks have been used to examine unprofessional behaviour, and how do these vary across different regions in the Global South?
3. What is the reported prevalence of unprofessional behaviour in various health professions education programmes in the Global South?
4. In what settings (clinical, academic, or other) are unprofessional behaviours most frequently encountered?
5. How are instances of unprofessional behaviour reported, addressed, and managed within these contexts?

### Identifying relevant studies

To ensure a comprehensive and systematic approach to literature retrieval, we collaborated with an expert health sciences librarian to design and refine a robust search strategy. This strategy combined both controlled vocabulary (e.g., MeSH terms) and free-text keywords, structured using Boolean operators (AND/OR), to capture the breadth of literature related to unprofessional behaviour in health professions education within the Global South. For instance, search strings such as (“unprofessional behaviour” OR “professional misconduct” OR “professionalism lapse”) AND (“health professions education” OR “medical students” OR “nursing education” OR “occupational therapy”) were tailored to the indexing system for each database (Supplementary Table 11).

Searches were conducted across multiple electronic databases, including PubMed/MEDLINE, Scopus, Web of Science, EBSCOhost (Academic Search Complete, Health Source, and PsycINFO) from their inception to June 2025, and were supplemented by Google Scholar to ensure the inclusion of all relevant research. Additional manual searches of the reference lists of all included full-text articles were conducted to identify additional eligible studies.

This scoping review was limited to studies published in English due to the linguistic capacities of the research team and the predominance of English in scholarly publishing. To ensure academic rigour and reliability, only peer-reviewed publications that employed primary study designs were included, thereby excluding unpublished reports, opinion pieces, and editorials that did not meet the standards of formal academic research.

### Study selection and eligibility criteria

All citations identified through the search process were imported into EndNote reference management software, where duplicate records were removed using the built-in “Find Duplicates” feature. The study selection process was carried out in two phases: [1] title and abstract screening, followed by [2] full-text review, based on a set of predefined inclusion and exclusion criteria.

To facilitate consistent screening, a structured selection tool was developed using Google Forms and piloted prior to full implementation. Two reviewers (DK and LP) independently screened the titles and abstracts, classifying them as either “include” or “exclude.” Any discrepancies during this phase were discussed and resolved by consensus. For studies that passed the initial screening, full-text articles were retrieved and independently assessed against the eligibility criteria by both reviewers. In cases where consensus could not be reached, a third reviewer (JvW) was consulted to make the final decision. The overall selection process was documented using the PRISMA flow diagram, which serves to ensure methodological transparency and traceability of study inclusion.

#### Inclusion criteria

This scoping review included studies:

- involving students, educators, or practitioners in health professions training programmes (e.g., medicine, nursing, occupational therapy, etc.).
- that focus on unprofessional behaviour in the context of health professions education.
- reporting descriptions, conceptions, or theoretical frameworks related to unprofessionalism.
- reporting prevalence or types of unprofessional behaviour.
- conducted in countries categorised as part of the Global South.
- Observational studies.
- Peer reviewed articles.
- English language publications.

#### Exclusion criteria

- Studies set exclusively in high-income countries.
- Articles unrelated to educational or training contexts.
- Opinion pieces or commentaries lacking empirical or conceptual analysis.
- Other review articles.

### Charting the data

Data extraction was carried out using a structured spreadsheet, which was initially pilot tested on three of the included studies to evaluate its effectiveness in capturing the necessary information relevant to the review objectives. Based on the feedback from this pilot, the extraction tool was refined to enhance clarity and comprehensiveness. Following this, two reviewers independently reviewed the full-text articles and extracted the required data.

The data extraction process combined deductive and inductive approaches [18], allowing for both structured retrieval of predefined variables and the identification of emerging insights. The finalised data extraction form included the following elements:

- Author(s), year of publication, and study location.
- Discipline(s) and participant group (e.g., students, educators).
- Study design and methods.
- Descriptions and conceptions of unprofessional behaviour.
- Theoretical or conceptual frameworks employed.
- Reported prevalence and types of unprofessional behaviour.
- Context in which unprofessional behaviours occurred (e.g., academic, clinical).
- Reporting and response mechanisms.
- Key outcomes and recommendations.

This dual approach ensured that both anticipated and unanticipated themes relevant to the research questions were systematically captured.

### Collating, summarising, and reporting the results

Findings were synthesised using both quantitative (descriptive statistics) and qualitative (narrative thematic analysis) approaches [19]. Data were organised according to the review objectives and presented under the following thematic headings:

- Descriptions and Conceptions.
- Stated or Implied Theoretical and Conceptual Frameworks.
- Prevalence and Patterns of Unprofessional Behaviour.
- Contextual Factors and Settings.
- Reporting and Management Mechanisms.

## Results

### Study selection

**Fig. 1:**
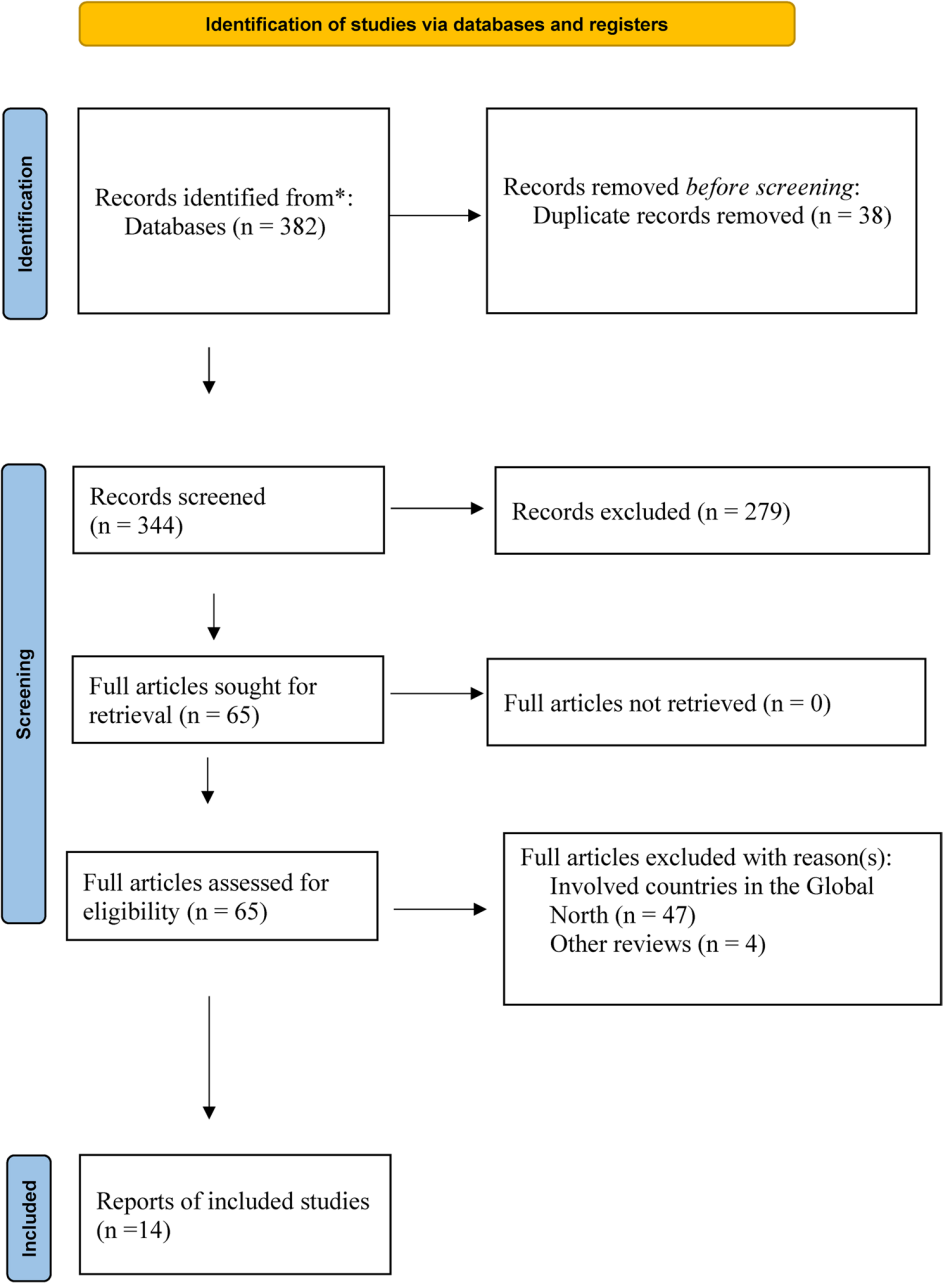
PRISMA 2020 flow diagram

A total of 382 titles and abstracts were initially identified through database searches. After removing duplicates and excluding records that did not meet the inclusion criteria, 14 studies were selected for data extraction and included in the narrative synthesis (Fig. 1).

### Characteristics of the included studies

Table 2 provides a detailed summary of the characteristics of the 14 included studies. The studies span 10 countries, with the majority conducted in the United Arab Emirates (21.4%) [20–22], followed by Saudi Arabia (14.3%) [7, 23, 24], and Thailand (14.3%) [25, 26] and other countries, each contributing 7.1% of the total studies. The publication years range from 2004 to 2024, with the highest number of studies were published in 2017, 2020, and 2023 (14.3% each).

**Table 2:** Characteristics of the included studies (*N* = 14)

Characteristics	Frequency (%)
Study country	
United Arab Emirates(20–22)	3 (21.4)
Saudi Arabia(7, 23, 24)	2 (14.3)
Thailand(25, 26)	2 (14.3)
India(27)	1 (7.1)
Egypt(28)	1 (7.1)
Kenya(30)	1 (7.1)
Iran(14)	1 (7.1)
South Africa(8)	1 (7.1)
South Korea(29)	1 (7.1)
Sri Lanka(12)	1 (7.1)
***Year of publication***	
2004	1 (7.1)
2011	1 (7.1)
2015	1 (7.1)
2016	1 (7.1)
2017	2 (14.3)
2018	1 (7.1)
2019	1 (7.1)
2020	2 (14.3)
2022	1 (7.1)
2023	2 (13.3)
2024	1 (7.1)
Study design	
Cross-sectional study(8, 20–28)	10 (71.5)
Qualitative study(12, 14, 29)	3 (21.4)
Mixed-methods study(30)	1 (7.1)
Study population	
Medical students(7, 8, 12, 20, 22–25, 27, 28)	9 (64.4)
Residents(26, 29)	2 (14.3)
Medicine, Pharmacy, and Nursing student(21)	1 (7.1)
Clinicians, medical students and allied health students(30)	1 (7.1)
Clinical faculty members and medical students(14)	1 (7.1)
Study settings	
Academic (Preclinical) and clinical(7, 14, 20, 22–25, 28)	7 (50.0)
Clinical only(8, 12, 26, 29)	4 (28.6)
Preclinical only(21, 27)	2 (14.3)
Clinical and surgical training environments(30)	1 (7.1)

In terms of study design, the majority (71.5%) were cross-sectional studies [8, 20–28], while qualitative studies accounted for 21.4% [12, 14, 29], and a smaller proportion was mixed-methods (7.1%) [30]. The majority of study participants were medical students(64.4%) [7, 8, 12, 20, 22–25, 27, 28], followed by residents (14.3%) [26, 29]. Smaller groups included multidisciplinary student cohorts from Medicine, Pharmacy, Nursing [21], clinicians and medical students [30], as well as clinical faculty members and medical students [14] each representing 7.1% of the study populations.

Regarding study settings, most studies were conducted across academic (preclinical) and clinical settings (50.0%) [7, 14, 20, 22–25, 28], with others focusing solely on clinical environments (28.6%) [8, 12, 26, 29], preclinical settings (14.3%) [21, 27], and a clinical and surgical training environment (7.1%) [30].

## Summary of Findings

### Unprofessional Behaviour Definitions

The 14 included studies provided diverse definitions of unprofessional behaviour (Table 3). Through thematic analysis, five key themes emerged: Academic Dishonesty and Integrity Violations, Bullying and Harassment, Clinical and Ethical Misconduct, Disrespect and Power Abuse, and Neglect of Professional Responsibilities.

**Table 3:** Types of unprofessional behaviour

Theme	Definition	References
Academic dishonesty and integrity violations	Includes cheating, plagiarism, falsification of records, research fraud, and misconduct in medical education. Violations extend to unethical academic practices such as signing attendance for absent peers and data fabrication.	Abdulrahman et al. [20], Rizk and Elzubeir [22], and Salih et al. [24]
Bullying and Harassment	Bullying encompasses persistent degrading, intimidating, malicious, or offensive behaviour that undermines confidence and self-esteem. It includes verbal, physical, emotional, sexual, and cyberbullying, often involving an imbalance of power. Harassment extends to discrimination based on gender, ethnicity, and social status.	AlMulhim et al. [23], Ibrahim et al. [21], Elghazally et al. [28], Naothavorn et al. [25], Tungsupreechameth et al. [26].
Clinical and Ethical Misconduct	Unprofessional behaviour in clinical practice includes patient mistreatment, lack of informed consent, confidentiality breaches, and negligence. Ethical misconduct extends to conflict of interest, research misconduct, bribery, and substance misuse.	Chang et al. [29], Rizk and Elzubeir [22], and Vivian et al. [8]
Disrespect and Power Abuse	Unprofessional behaviours related to power dynamics include verbal abuse, discrimination, hierarchical mistreatment, and lack of respect for colleagues, patients, and cadavers. Gendered power struggles and hierarchical exploitation in medical education and practice have also been identified.	Karunakaran et al. [27], Shaw et al. [12], Tabatabaei et al. [14], and Vivian et al. [8].
Neglect of Professional Responsibilities	Unprofessional behaviour includes irresponsibility, failure to consult colleagues, intellectual apathy, and resistance to learning. It also involves neglecting professional duties, failing to follow medical protocols, and lacking accountability in patient care.	Ojuka et al. [30], Tabatabaei et al. [14]

#### Academic Dishonesty and Integrity Violations

 Unprofessional behaviour in academic settings primarily involves acts of dishonesty, such as cheating, plagiarism, falsification of records, and research fraud [7, 20, 22, 24]. Violations extend to unethical academic practices, including signing attendance for absent peers and fabricating data in research [7, 20, 22, 24]. These behaviours compromise the integrity of medical education and professional development, ultimately affecting clinical competence and patient safety [7, 20, 22, 24].

#### Bullying and Harassment

 Bullying and harassment are significant unprofessional behaviours in both academic and clinical settings, characterised by persistent, degrading, intimidating, or offensive actions [21, 23, 25, 26, 28]. Various forms of bullying include verbal (insults, name-calling), physical (pushing, hitting), emotional (social exclusion, discrimination), sexual (harassment, molestation), and cyberbullying (online threats, offensive messages) [21, 23, 25, 26, 28]. Power imbalances often exacerbate these behaviours, impacting students, trainees, and professionals in medical and healthcare education [21, 23, 25, 26, 28].

#### Clinical and Ethical Misconduct

 Unprofessional behaviour in clinical practice involves violations of ethical and medical standards, including patient mistreatment, failure to obtain informed consent, breaches of confidentiality, and clinical negligence [7, 8, 22, 29]. Ethical misconduct extends to conflict of interest, research fraud, bribery, and substance misuse, all of which can compromise patient care and professional trust [7, 8, 22, 29]. These behaviours undermine the integrity of medical practice and can result in serious legal and professional consequences [7, 8, 22, 29].

#### Disrespect and Power Abuse

 Disrespect in medical education and practice includes verbal abuse, discrimination, hierarchical mistreatment, and a lack of respect for colleagues, patients, and cadavers [8, 12, 14, 27]. This theme highlights power struggles, particularly gendered discrimination and hierarchical exploitation, where junior medical professionals and students are subjected to mistreatment by superiors [8, 12, 14, 27]. Such behaviours create toxic learning and working environments, impacting professionalism and ethical conduct [8, 12, 14, 27].

#### Neglect of Professional Responsibilities

 Neglect of professional responsibilities is an unprofessional behaviour characterised by irresponsibility, failure to consult colleagues, intellectual apathy, and resistance to learning [14, 30]. It also includes neglecting professional duties, failure to follow medical protocols, and lack of accountability in patient care [14, 30]. These behaviours compromise healthcare delivery and patient safety, highlighting the need for accountability and adherence to professional medical standards [14, 30].

### Concepts of Unprofessional Behaviour

 Among the 14 included studies, 11 studies explored various concepts of unprofessional behaviour (Table 4). Through thematic analysis, five key themes emerged: Academic Dishonesty and Ethical Lapses; Bullying, Mistreatment, and Psychological Impact; Hierarchical Power Dynamics and Cultural Norms; Professionalism and Role Modelling in Medical Education; and Gender Bias and Professional Identity Development.

**Table 4:** Concepts of unprofessional behaviour

**Theme**	**Summary of concept**	**References**
Academic Dishonesty and Ethical Lapses	Academic dishonesty is viewed as a fundamental threat to professional ethics in medicine, with potential long-term consequences on patient care. While students recognise professional misconduct, ethical breaches in doctor–patient interactions are often considered more severe than academic dishonesty. Cultural and institutional expectations shape students' attitudes toward ethical decision-making and professional conduct.	Abdulrahman et al.[20] Rizk and Elzubeir[22], and Salih et al.[24]
Bullying, Mistreatment, and Psychological Impact	Bullying is prevalent in medical education and is often normalised within hierarchical training structures. It disproportionately affects students at lower levels, especially females, and leads to significant stress, anxiety, and depression. Mistreatment, particularly in higher academic years, remains underreported due to fears of retaliation	AlMulhim et al.[23], Ibrahim et al.[21], Elghazally et al.[28], and Naothavorn et al.[25]
Hierarchical Power Dynamics and Cultural Norms	Unprofessional behaviour is often shaped by hierarchical medical structures that discourage reporting, reinforce rigid seniority-based respect, and contribute to the mistreatment of junior doctors. Cultural influences also play a role, as certain behaviours are tolerated based on institutional and societal norms. Students and junior healthcare workers frequently report experiencing mistreatment due to these rigid hierarchies.	Chang et al.[29], Ojuka et al.[30], and Shaw et al.[12]
Professionalism and Role Modelling in Medical Education	Professionalism can be introduced early in medical training, particularly through faculty role modelling and structured training programs. Anatomy education and dissection halls provide early opportunities to instil respect, accountability, and ethical responsibility. Transitioning into clinical training further enhances students' awareness of professionalism standards.	Karunakaran et al.[27].
Gender Bias and Professional Identity Development	Gendered professionalism lapses are recognised but often go unchallenged due to cultural and institutional hierarchies. Gender biases affect students' professional identity development, and in some cases, students reproduce discriminatory behaviours they observe during training.	Shaw et al.[12]

#### Academic Dishonesty and Ethical Lapses

 Academic dishonesty is widely recognised as a fundamental threat to professional ethics in medicine, with potential long-term consequences for patient care [20, 22, 24]. While students acknowledge professional misconduct, breaches in doctor–patient interactions are often perceived as more serious than academic dishonesty [20, 22, 24]. Additionally, cultural and institutional expectations shape students' attitudes toward ethical decision-making and professional conduct [20, 22, 24].

#### Bullying, Mistreatment, and Psychological Impact

 Bullying is a prevalent and often normalised issue in medical education, particularly within hierarchical training structures [21, 23, 25, 28]. It disproportionately affects students at lower levels, especially females, leading to significant psychological distress such as anxiety, depression, and burnout [21, 23, 25, 28]. Mistreatment is most common in higher academic years and remains underreported due to institutional norms and fear of retaliation [21, 23, 25, 28].

#### Hierarchical Power Dynamics and Cultural Norms

 Unprofessional behaviour is often shaped by rigid medical hierarchies that discourage reporting and reinforce seniority-based respect, leading to the mistreatment of junior doctors and students [12, 29, 30]. Cultural factors also play a role, as institutional norms often tolerate certain unprofessional behaviours [12, 29, 30]. Junior healthcare workers and students frequently report experiencing mistreatment due to their subordinate positions, further perpetuating these hierarchical inequities [12, 29, 30].

#### Professionalism and Role Modelling in Medical Education

 Professionalism in medicine can be effectively introduced early in medical training through faculty role modelling and structured training programs [7, 27]. Anatomy education and dissection halls provide early opportunities to instil respect, accountability, and ethical responsibility [7, 27]. Additionally, transitioning into clinical training increases students' awareness of professionalism standards, reinforcing the importance of ethical behaviour in medical practice [7, 27].

#### Gender Bias and Professional Identity Development

 Gendered professionalism lapses, taking the form of impinging on patient dignity, safety, consent dilemmas, and female student abuse, are widely recognised but often go unchallenged due to institutional hierarchies and cultural norms [12]. Gender biases influence students' professional identity development, and some students, females more than males, inadvertently reproduce discriminatory behaviours they observe in medical training [12]. This phenomenon highlights the need for addressing systemic biases to promote a more equitable and professional medical education environment [12].

### Prevalence of Unprofessional Behaviour

 Unprofessional behaviour is widely prevalent across different aspects of medical education, residency training, and clinical practice, as reported in 11 of the 14 included studies. The findings highlight high rates of academic dishonesty, bullying, mistreatment, ethical violations, and professionalism lapses among medical students, residents, and faculty. Below is a narrative synthesis of the key findings across the identified themes.

#### Academic Dishonesty and Ethical Violations

 Academic dishonesty is a significant issue among medical students, with 83% admitting to committing at least one act of misconduct, including proxy attendance (42%), copying from record books (12%), and attempting to obtain exam questions (23%)(20). While students widely recognised unprofessional behaviours as inappropriate, only 5.7%–6.8% admitted to engaging in or considering such behaviours (22). Furthermore, students were less likely to report academic dishonesty compared to clinical misconduct, with only 15.5% willing to report unprofessional acts (7, 24). The severity of ethical violations varied, with failure to consult specialists being perceived as the most serious misconduct (40.9%), while plagiarism was considered the least serious (6.8%)(22).

#### Bullying, Mistreatment, and Psychological Impact

 Bullying and mistreatment are highly prevalent in medical education, with 49%–74.5% of students reporting experiences of mistreatment [23, 25]. Verbal bullying was the most reported form, affecting 44.4% of students, followed by cyberbullying (23.8%) and emotional bullying (19.8%)[21]. Sexual harassment and physical abuse, although reported at lower rates (≤7.9%), were still present [23, 25, 26]. Mistreatment was particularly common in clinical years, where students faced excessive workloads, exclusion from learning opportunities, and tasks beyond their competence [25, 26]. Female students were more likely to experience verbal and behavioural bullying, while male students reported higher rates of physical and written bullying [28].

#### Hierarchy and Power Abuse in Medical Training

 Medical students and residents frequently witnessed unprofessional behaviours linked to hierarchical power dynamics, with residents reporting 3 to 18 incidents per individual [29]. Common unprofessional acts included failing to fulfil patient care duties, fabricating medical test results, and verbal or physical abuse of junior doctors. The rigid medical hierarchy discouraged reporting such behaviours due to fears of retaliation and cultural norms normalising mistreatment [12, 30]. Female medical students particularly reported discrimination and harsher treatment compared to their male counterparts[12]. The normalisation of mistreatment within medical education contributed to a culture where students reproduce discriminatory behaviours they observe [12]. 

## Professionalism lapses in anatomy education and clinical practice

A significant proportion of faculty members raise the importance of addressing the lack of respect for cadavers and failure to follow ethical guidelines in the anatomy labs (75%) [27]. While 100% of faculty agreed on the necessity of professionalism instruction, only 65% strictly enforced ethical guidelines, highlighting gaps in professionalism education [27]. Additionally, in clinical practice, 71% of medical students witnessed patient rights abuses, with 38% reporting physical abuse and 37% reporting verbal abuse [8].

### Perceptions and sanctions for unprofessional behaviour

Faculty members generally perceived unprofessional behaviour as more problematic than students did, with 595 instances of unprofessional behaviour identified compared to 490 professional behaviour cases [14].

### Reporting mechanisms for unprofessional behaviour

The reporting of unprofessional behaviour in medical education and clinical practice is highly inconsistent and largely ineffective, with most students and trainees choosing not to report incidents due to fear of retaliation, cultural barriers, and lack of institutional support. Across multiple studies, reporting mechanisms were found to be inadequate or underutilised, with many institutions lacking formal systems for addressing unprofessional behaviour. Below is a narrative synthesis of the key findings.

#### Low reporting rates and fear of retaliation

A significant number of students and trainees do not report unprofessional behaviour. In multiple studies, over 90% of students did not report bullying or mistreatment [25, 28]. Similarly, only 16.4% of mistreated residents formally reported their experiences, with 52.5% fearing negative career consequences [26]. Sexual harassment was particularly underreported, with students feeling reluctant to disclose such incidents due to cultural and social pressures [23].

#### Lack of formal reporting structures

Many institutions lack structured or effective formal mechanisms for reporting unprofessional behaviour. In some cases, no formal system existed at all, leaving students and trainees to handle incidents informally or through faculty mentorship rather than disciplinary measures [27, 29, 30]. Among those who reported incidents, only 20% were satisfied with the reporting process [25]. Additionally, 65% of residents were unaware of any institutional policies on mistreatment, highlighting a critical gap in organisational accountability [26].

#### Alternative reporting channels and informal responses

When students and trainees did report incidents, they often turned to family, friends, or advisors rather than institutional authorities. Only 14.3% of bullied students reported incidents to student affairs, while 27% informed a family member or advisor, and 19% confided in a friend [21]. Similarly, 91.3% of bullied students did not report incidents to faculty, with only 8.7% discussing their experiences informally [28]. Many students also resorted to self-reported surveys rather than institutional reporting channels [20].

#### Cultural and institutional barriers to reporting

Cultural and institutional norms discourage students from reporting. Students feared jeopardising their future careers if they formally reported incidents, leading to self-censorship and passive acceptance of misconduct [12]. Although some institutions had formal reporting mechanisms in place, these were seldom used, as students feared potential negative repercussions for lodging complaints [14].

#### Perceptions of professionalism and willingness to report

Despite widespread acknowledgement of unprofessional behaviour, few students were willing to report incidents, with only 15.5% expressing a willingness to do so [24]. Additionally, while 72.7% of respondents believed doctors should report unprofessional colleagues, only 36.4% supported dismissal as a penalty for serious misconduct, indicating hesitancy in enforcing disciplinary actions [22]. These findings highlight critical gaps in reporting mechanisms for unprofessional behaviour.

### Management and responses to unprofessional behaviour

Efforts to address and manage unprofessional behaviour in medical education and clinical settings remain largely insufficient, with limited punitive actions, lack of structured policies, and inadequate institutional responses. However, several studies highlight potential interventions, including formal professionalism training, structured mentorship, and anonymous reporting mechanisms. Below is a narrative synthesis of the key findings.

#### Limited institutional policies and formal responses

Many institutions lack structured approaches to addressing academic misconduct and bullying. In medical schools, punitive actions against academic dishonesty are minimal, with no clear policies in place to prevent unprofessional behaviour [20, 28]. Similarly, bullying and mistreatment are widely underreported, and institutions do not have formal mechanisms to protect students or enforce corrective measures [25]. Faculty often rely on verbal instructions and informal mentorship, rather than structured curricula to instil professionalism [27].

#### Professionalism training and institutional interventions

Several studies emphasize the importance of integrating professionalism training into medical education. Formal ethics courses, faculty mentorship, and structured assessments of professionalism are recommended to align faculty and student expectations [14, 24]. In response to widespread mistreatment, the Mistreatment Awareness Program (MAP) was implemented, including educational posters and video-based discussions, which led to a decline in bullying incidents [26].

#### Barriers to Reporting and Cultural Norms

Despite recognising unprofessional behaviour, students and residents often remain passive observers due to fear of retaliation, cultural barriers, and a lack of institutional support. While students expressed shock and discomfort, they rarely intervened in cases of mistreatment, instead resorting to informal consoling of victims [8]. In some cases, students engaged in subtle resistance, such as documenting unethical practices in patient charts, though these actions had a limited impact [12]. Additionally, cultural norms influenced students’ perceptions of penalties, with many preferring remedial ethics training over dismissal for misconduct [22].

#### Recommendations for Strengthening Institutional Policies

To effectively manage unprofessional behaviour, several studies advocate for comprehensive institutional reforms. Recommended strategies include implementing awareness programs to educate students and faculty on professionalism [23]; establishing anonymous reporting systems to encourage victims to disclose incidents [28, 29]; enforcing stricter penalties for unprofessional conduct, including dismissal for repeated or severe offences [21]; introducing mentorship programmes to provide ethical guidance and professional role modelling [30]; and delivering gender-sensitivity training to address biases and foster inclusive learning environments [12]. These findings highlight significant gaps in the management of unprofessional behaviour.

## Discussion

This scoping review explored how unprofessional behaviour is conceptualised, theorised, and addressed within health professions education (HPE) in the Global South. The findings illustrate that professionalism is a dynamic, negotiated, and contested construct rather than a fixed one. The findings highlight the contextual complexity of unprofessionalism, showing how cultural norms, institutional hierarchies, and resource constraints shape the way such behaviours are understood, experienced, and managed. Five key discussion points aligned with the research questions are elaborated below.

### Conceptions of Unprofessional Behaviour

In the literature from the Global South, definitions and descriptions of unprofessional behaviour vary considerably, reflecting differences in institutional priorities and cultural norms. Commonly cited forms of unprofessionalism include academic dishonesty (e.g., cheating, plagiarism, falsification of records) (20, 24), bullying and harassment (23, 25), ethical violations in clinical practice (8, 29), disrespect and power abuse (12, 14), and neglect of professional responsibilities (30). These behaviours are often shaped by the hidden curriculum, institutional silence, and the implicit cultural codes governing professional conduct. The variability in definitions complicates comparative research and underscores the need for context-sensitive understandings of professionalism.

### Theoretical Frameworks and Regional Variations

The review found limited use of explicit theoretical frameworks to analyse unprofessional behaviour. However, several studies implicitly drew on key conceptual lenses. Professional identity formation and role modelling were commonly referenced in studies from India and Saudi Arabia, especially in preclinical education settings such as anatomy laboratories (7, 27). In contrast, studies from South Africa, Iran, and Sri Lanka highlighted how hierarchical power structures and gendered norms influence behaviour, aligning with critical and feminist theories (8, 12, 14). Southeast Asian contexts, such as Thailand and South Korea, revealed how cultural norms of deference and silence contribute to the normalisation of mistreatment (25, 29). These regional variations indicate the need for theoretical pluralism and the development of regionally grounded frameworks that reflect local values, power relations, and professional expectations. The lack of explicit theoretical framing in the included studies limits the field’s ability to explain why these behaviours persist, how they are reproduced intergenerationally, and how they are legitimised institutionally.

### Prevalence of Unprofessional Behaviour

The review confirms that unprofessional behaviour is highly prevalent in health professions education programmes in the Global South. However, the reported prevalence should be interpreted with caution, as the included studies vary in their methodological approaches, measurement instruments, contexts, and timeframes. Consequently, the findings reflect the scope and seriousness of professionalism challenges rather than providing directly comparable prevalence estimates. Among the studies reviewed, academic misconduct was reported by up to 83% of students, including practices such as proxy attendance and data fabrication (20). Mistreatment and bullying were reported by 49% to 74.5% of students, with verbal bullying being most common, followed by emotional and cyberbullying (21, 28). Clinical ethical violations, including patient mistreatment and breaches of confidentiality, were also frequently observed (8). However, the true prevalence may be underestimated due to widespread underreporting and the lack of standardised assessment tools.

### Settings Where Unprofessional Behaviour is Most Frequently Encountered

Clinical environments emerged as the most common settings in which unprofessional behaviour occurred. These included patient care sites, hospital wards, and residency programmes where students and junior staff are vulnerable to mistreatment, hierarchical abuse, and ethical lapses (8, 26, 29). Academic settings, particularly preclinical classrooms and assessment areas, were linked to incidents of academic dishonesty and lapses in professionalism within anatomy laboratories (22, 27). Transitions from academic to clinical environments were identified as particularly vulnerable periods, where inconsistent role modelling and ambiguous expectations heightened risks (7, 12). The transitional nature of professional learning suggests a need for early and continuous reinforcement of ethical standards across educational phases.

### Reporting, Addressing, and Managing Unprofessional Behaviour

The review found that unprofessional behaviour is significantly underreported . Over 90% of students in some studies did not report mistreatment, while among residents, only 16.4% formally reported such experiences (25, 26). Many institutions lack formal reporting systems, and where they exist, students often bypass them in favour of informal discussions with peers, family, or mentors (21, 27). Institutional responses, when they occur, are often limited to verbal warnings or informal mentorship. However, a few studies reported promising interventions, such as the Mistreatment Awareness Program in Thailand, which reduced bullying through awareness posters and video-based discussions (26). These findings point to an urgent need for structured, confidential, and culturally responsive reporting and remediation systems, along with faculty development and institutional accountability.

### Power, Gender and Institutional Protection

Socio-political environments can create conditions that enable unprofessional behaviour to persist if left unchecked. Our findings illustrate that mistreatment can become an integral part of the clinical experience for students, often stemming from entrenched professional hierarchies and political-cultural expectations (26). Gender influences perceptions of professionalism, with women often facing unique forms of harassment and being regarded as less deserving of protection (12, 23). Trainees frequently fear professional repercussions if they report incidents, leading to under-reporting (8). If students are unaware of or where institutions do not have formal mechanisms to protect complainants, it creates fertile ground for unprofessional behaviour to be normalised (25). Institutional culture can give the impression of acceptance of mistreatment through acquiescence unless it challenges powerful hierarchies.

### A model for unprofessional behaviour

**Fig. 2:**
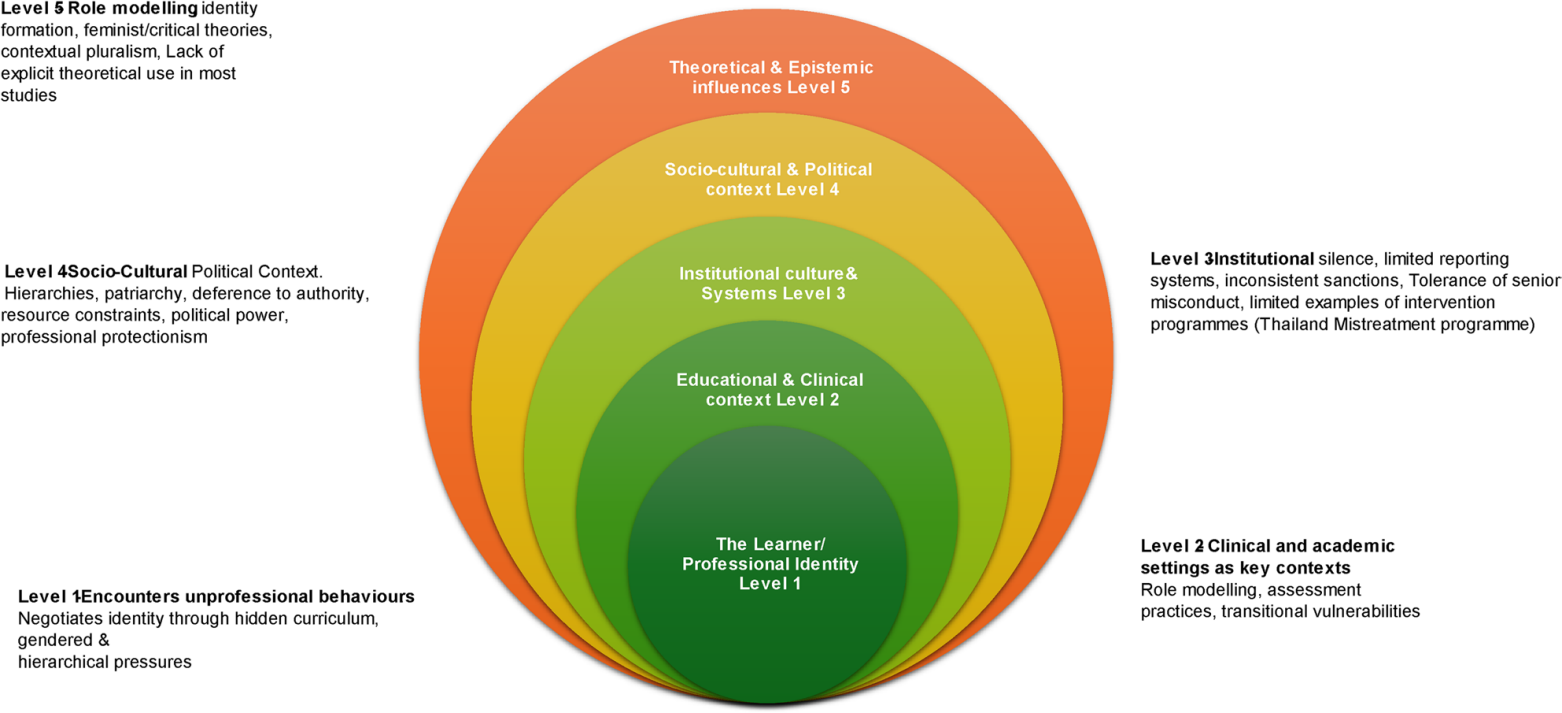
Contextually Mediated Professionalism in Health Professions Education: A Relational Model for the Global South

Based on our findings, we present this framework, which illustrates how unprofessional behaviour arises and is sustained across interacting layers — from individual identity formation through institutional systems to socio-cultural and political contexts. It emphasizes the dynamic nature and influences that shape professionalism (Fig. 2).

### Strengths and limitations of this scoping review

This scoping review demonstrates several strengths and limitations. A key strength lies in its systematic use of Arksey and O’Malley’s methodological framework, refined by Levac et al., and reported according to PRISMA-ScR guidelines, which enhances transparency and replicability. The review also benefits from a comprehensive and librarian-assisted search strategy across multiple major databases, supplemented by manual searches, ensuring wide coverage of relevant literature. Another strength is the inclusion of diverse study populations (students, residents, faculty, and clinicians) from multiple Global South countries, which provides a nuanced understanding of unprofessional behaviour across varied cultural and institutional contexts.

Limitations include the restriction to English-language publications, which may have excluded important evidence from non-English-speaking Global South countries. The review also synthesised only 14 studies, highlighting a sparse evidence base and limiting the generalisability of findings. Additionally, the predominance of cross-sectional studies, lack of consistent theoretical frameworks, and reliance on self-reported data may introduce bias and underreporting, thereby constraining the depth of insights into prevalence and causality. The review did not include grey literature, institutional policy, or internal disciplinary records; it may therefore underestimate the scale of institutional abuse or tolerance and the extent of local attempts at reform. Furthermore, we did not assess the quality or the risk of bias of included studies, which can lead to the inclusion of studies with weak or low-quality evidence. The reported prevalence figures originate from individual studies conducted across different countries, time periods, and using diverse instruments, rather than representing a pooled estimate for the Global South. However, this approach aligns with PRISMA-ScR guidelines, which emphasize mapping the breadth of available evidence rather than statistically aggregating data. Despite these limitations, the findings are useful for informing context-specific policy, curriculum reforms, and institutional interventions aimed at strengthening professionalism training and addressing unprofessional behaviour within health professions education in the Global South.

## Conclusions

The review maps existing published evidence rather than evaluating interventions therefore recommendations for reform are proposed directions rather than evidence-based solutions. The findings of this scoping review illustrate that unprofessional behaviour in health professions education in the Global South occurs broadly, is weakly conceptualised, and is poorly reported on. The study highlights the need for context-specific conceptions, theoretical engagement, and institutional reforms that reflect the realities of educational and clinical training environments in low- and middle-income countries. It underscores the point that theoretical and conceptual development should emerge from within or be related to low-middle-income contexts, rather than being imported wholesale from the Global North professional discourses. Future research should focus on developing culturally grounded professionalism frameworks, evaluating the impact of reporting systems, and exploring longitudinal interventions that support professional identity formation across training trajectories.

## Supplementary Information

Supplementary Material 1.

Supplementary Material 2.

## Data Availability

Data reported in this article are presented in the manuscript and as additional supporting supplementary files. Supplementary Table 1: Literature searchesSupplementary Table 2: PRISMA-ScR Checklist.
