# Supplementary material for: Health professions education and unprofessional behaviour in the global south: a scoping review of conceptions, theoretical frameworks, and prevalence
Source: BMC Med Educ. 2025 Dec 29;25:1733. doi: 10.1186/s12909-025-08318-w (PMC12751322; doi:10.1186/s12909-025-08318-w)
Supplement: Supplementary file 1 — Supplementary Material 1. [file 12909_2025_8318_MOESM1_ESM.docx]

**Supplementary Material**

**Supplementary Table 1:** Literatures searches

| **Date** | **Database** | **Search string** | **Yield** | **Titles eligible** |
| --- | --- | --- | --- | --- |
| 20/06/2025 | PubMed | Search # 1: "students, medical"[MeSH Terms] OR ("students"[All Fields] AND "medical"[All Fields]) OR "medical students"[All Fields] OR ("medical"[All Fields] AND "student"[All Fields]) OR "medical student"[All Fields] OR (("occupational therapy"[MeSH Terms] OR ("occupational"[All Fields] AND "therapy"[All Fields]) OR "occupational therapy"[All Fields]) AND ("student s"[All Fields] OR "students"[MeSH Terms] OR "students"[All Fields] OR "student"[All Fields] OR "students s"[All Fields])) OR (("health occupations"[MeSH Terms] OR ("health"[All Fields] AND "occupations"[All Fields]) OR "health occupations"[All Fields] OR ("health"[All Fields] AND "profession"[All Fields]) OR "health profession"[All Fields]) AND ("student s"[All Fields] OR "students"[MeSH Terms] OR "students"[All Fields] OR "student"[All Fields] OR "students s"[All Fields]))  Search # 2: "unprofessional behavior"[All Fields] OR "unprofessional conduct"[All Fields] OR "professional misconduct"[All Fields] OR "professional behavior"[All Fields] OR "ethical behavior"[All Fields] OR "professionalism"[All Fields]  Search # 3: "theoretical framework"[All Fields] OR "concept"[All Fields] OR "definition"[All Fields] OR "prevalence"[All Fields] OR "incidence"[All Fields] OR "reporting"[All Fields]  Search # 4: "health sciences education"[All Fields] OR "medical education"[All Fields] OR "Health Professions education"[All Fields] OR "clinical training"[All Fields] OR "healthcare training"[All Fields]  Search # 5: 1 AND 2 AND 3 AND 4 | 251,660  16,368  2,743,342  261,918  360 | 121 |
| 25/06/2025 | Web of Science | medical student OR Occupational therapy student OR health profession student AND unprofessional behaviour <https://www.webofscience.com/wos/woscc/summary/0dd0f5d8-4ecd-46c8-8ce5-0911a0c26249-0156dc86e9/relevance/1> | 3708 | 65 |
| 27/06/2025 | SCOPUS | TITLE-ABS-KEY (health AND profession AND student AND clinical AND education OR training AND unprofessional). | 2631 | 45 |
| 28/06/2025 | ESCOhost (Academic search complete, ERIC, PsychoINFO, and Health Source: Nursing/Academic Edition | medical student OR Occupational therapy student OR health profession student AND unprofessional behaviour | 107 | 18 |
| 30/06/2025 | Google Scholar | medical student OR Occupational therapy student OR health profession student AND unprofessional behaviour behavior OR unprofessional conduct OR professional misconduct OR professional behavior OR ethical behavior OR professionalism AND theoretical framework OR concept OR definition OR prevalence OR incidence OR reporting AND "health sciences education" OR "medical education" OR "Health Professions education" OR "clinical training" OR "healthcare training | 16,800 | (133 from first 1000) |
|  |  | Duplicates |  | 38 |
|  |  | No. Records for abstract screening |  | 344 |
